# Cardiovascular safety of linagliptin in type 2 diabetes: a comprehensive patient-level pooled analysis of prospectively adjudicated cardiovascular events

**DOI:** 10.1186/s12933-015-0215-2

**Published:** 2015-05-21

**Authors:** Julio Rosenstock, Nikolaus Marx, Dietmar Neubacher, Thomas Seck, Sanjay Patel, Hans-Juergen Woerle, Odd Erik Johansen

**Affiliations:** Dallas Diabetes and Endocrine Center at Medical City, Dallas, TX USA; Department of Internal Medicine I, University Hospital Aachen, Aachen, Germany; Boehringer Ingelheim, Biberach, Germany; Boehringer Ingelheim, Ingelheim, Germany; Boehringer Ingelheim, Bracknell, Berkshire, UK; Boehringer Ingelheim, Drengsrudbekken 8, 1373 Asker, Oslo, Norway

**Keywords:** Drug safety, Linagliptin, Adverse effects, Type 2 diabetes mellitus

## Abstract

**Background:**

The cardiovascular (CV) safety of linagliptin was evaluated in subjects with type 2 diabetes (T2DM).

**Methods:**

Pre-specified patient-level pooled analysis of all available double-blind, randomized, controlled trials, ≥12 weeks’ duration (19 trials, 9459 subjects) of linagliptin versus placebo/active treatment. Primary end point: composite of prospectively adjudicated CV death, non-fatal myocardial infarction, non-fatal stroke, and hospitalization for unstable angina (4P-MACE). Hospitalization for congestive heart failure (CHF) was also evaluated; adjudication of CHF was introduced during the phase 3 program (8 trials; 3314 subjects). 4P-MACE was assessed in placebo-controlled trials (subgroup of 18 trials; 7746 subjects). Investigator-reported events suggestive of CHF from 24 placebo-controlled trials (including trials <12 weeks’ duration, 8778 subjects) were also analyzed.

**Results:**

5847 patients received linagliptin (5 mg: 5687, 10 mg: 160) and 3612 comparator (glimepiride: 775, voglibose: 162, placebo: 2675); cumulative exposure, 4421.3 and 3254.7 patient-years, respectively. 4P-MACE incidence rates: 13.4 per 1000 patient-years, linagliptin (60 events), 18.9, total comparators (62 events); overall hazard ratio (HR), 0.78 (95% confidence interval [CI], 0.55–1.12). HR for adjudicated hospitalization for CHF (n = 21): 1.04 (0.43–2.47). For placebo-controlled trials, 4P-MACE incidence rates: 14.9 per 1000 patient-years, linagliptin (43 events), 16.4, total comparators (29 events); overall HR, 1.09 (95% CI, 0.68–1.75). Occurrence of investigator-reported events suggestive of CHF was low for linagliptin- (26 events, 0.5%; serious: 16 events, 0.3%) and placebo-treated (8 events, 0.2%; serious: 6 events, 0.2%) patients.

**Conclusions:**

Linagliptin is not associated with increased CV risk versus pooled active comparators or placebo in patients with T2DM.

## Background

Cardiovascular (CV) disease (CVD) is the major cause of premature death in patients with type 2 diabetes mellitus (T2DM), with approximately half of all deaths attributable to CV complications [1,2]. An important consideration in the treatment of T2DM is the management of CV risk factors, including hyperglycemia, being closely and independently associated with increased CV morbidity and premature mortality [3]. Evaluation of the CV effects of drugs used to lower blood glucose in T2DM is important, as illustrated by the controversy surrounding certain glucose-lowering drugs, such as rosiglitazone [4]. For other drugs, in particular the sulfonylureas (SUs) [5], the CV safety is contentious, with some reports suggesting an increased CV risk with certain SUs [6-8], while others report no increased risk [9,10]. Given the relationship between CV safety and T2DM and the uncertainty surrounding the CV risk of some therapies, the US Food and Drug Administration (FDA) requires evaluation of CV risk for new compounds being developed as therapies for T2DM [11]. Consequently, several trials are currently under way to evaluate the long-term CV outcomes of recently developed drugs. The first 2 placebo-controlled trials (SAVOR-TIMI 53 and EXAMINE) involving the dipeptidyl peptidase (DPP)-4 inhibitors saxagliptin and alogliptin, respectively, both reported a neutral effect on a composite of CV death (including fatal stroke and fatal myocardial infarction [MI]), non-fatal MI, and non-fatal stroke (3P-MACE) [12,13]. Neither SAVOR-TIMI 53 nor EXAMINE showed an increase in death from any cause versus placebo (hazard ratio [HR], 1.11, 95% confidence interval [CI], 0.96–1.27; *P* = .15, and HR, 1.03, 95% CI, 0.87–1.22; *P* = .72, respectively) or death from CV causes (HR, 0.79, 95% CI, 0.60–1.04; *P* = .10, and HR, 0.88, 95% CI, 0.71–1.09; *P* = .23, respectively). Of note, a statistically significant increased risk for hospitalization for congestive heart failure (CHF) associated with saxagliptin therapy was reported, and an analysis of data for alogliptin showed a non-significant HR above 1.0 [14]. A recent analysis of the end point of hospitalization for heart failure in SAVOR-TIMI 53 showed that saxagliptin therapy was associated with an increased risk of this event: more patients treated with saxagliptin (289, 3.5%) were hospitalized for heart failure compared with those allocated to placebo (228, 2.8%; HR, 1.27; 95% CI, 1.07–1.51; *P* = .007) [15]. This increase was greatest among patients with elevated levels of natriuretic peptides at baseline, previous heart failure, or chronic kidney disease. At present it remains unclear whether any potential increase in CHF is a causal effect.

Linagliptin is a selective and potent DPP-4 inhibitor with a xanthine-based molecular structure, and is indicated for the treatment of T2DM. The pharmacokinetic properties of linagliptin permit DPP-4 inhibition over 24 hours following once-daily dosing, and the drug is primarily excreted via the bile and gut [16,17]. In 2010, a pre-specified meta-analysis of CV events from 8 phase 3 studies was performed for linagliptin versus overall comparator-treated patients with T2DM [18]. All suspected CV events and fatalities were prospectively adjudicated by a blinded, independent clinical event committee (CEC). The primary end point was a composite of CV death, stroke, MI, and hospitalization for unstable angina (4P-MACE). Although the analysis was limited in terms of patient numbers (n = 5239) as well as total number of primary events (n = 34), this initial pooled CV analysis did not indicate any increased risk of CV events with linagliptin.

Following completion of several other phase 3 studies of linagliptin, contributing to a larger database than was previously available, the pooled analysis presented here provides a more comprehensive and up-to-date assessment of the CV safety profile of linagliptin versus comparator treatments in patients with T2DM. In addition, investigator-reported events suggestive of CHF are presented, using a pooling of 24 placebo-controlled trials.

## Methods

### Study selection

The first cohort analyzed included all available, completed phase 3 studies and 1 phase 2b study in the Boehringer Ingelheim (BI) project database for linagliptin (for study details, see Table 1) in which an independent adjudication of CV events (MACE-plus, defined as a composite end point consisting of the following adjudicated events: CV death, non-fatal MI, non-fatal stroke, and hospitalization for unstable angina) was conducted (the 8 trials with prospective independent adjudication of hospitalization for CHF are identified in Table 1). All studies were conducted in accordance with Good Clinical Practice guidelines and the principles of the Declaration of Helsinki. This pooled patient-level analysis was pre-specified and included only double-blind, randomized, controlled trials, lasting ≥12 weeks, evaluating linagliptin versus comparator (placebo or active treatment), with database lock-up to August 6, 2012. Data from all patients treated with a daily oral linagliptin dose of 5 mg or more were pooled into a total comparator cohort with a common linagliptin treatment group, and all randomized control treatments were combined into 1 common control treatment group (regardless of whether patients were treated with placebo or an active comparator, as per methodology suggested by the FDA [11]). As a sensitivity analysis, the impact on the primary end point 4P-MACE and on hospitalization for CHF in the placebo cohort alone (ie, only trials or parts of trials in which linagliptin was compared with placebo) was also evaluated. In an additional analysis, the occurrence of symptoms or adverse events (AEs) suggestive of heart failure was evaluated, based on newly updated results from 24 placebo-controlled trials (phases 1–3; 3 of which were <12 weeks in duration), involving 8778 patients (5488 linagliptin and 3290 placebo) (for details of the additional trials included, see Table 1). Investigator-reported AEs that may be indicative of possible heart failure were defined using the list of terms defined in the Medical Dictionary for Regulatory Activities (MedDRA 16.0; narrow standardized MedDRA queries [SMQ] cardiac failure). AE-reported terms coded to these specific MedDRA preferred terms were used to identify possible heart failure events.

**Table 1:** **Overview of linagliptin clinical trials included in the CV safety analysis of adjudicated events**

**Study number**	**Treatment**	**Patients**	**Background**	**Follow-up (weeks)**	**Reference/NCT number**
1218.15	Linagliptin 5 mg Placebo;	259 130	Pioglitazone	24	Gomis *et al.* [39]. NCT00641043
1218.16	Linagliptin 5 mg Placebo;	336 167	None	24	Del Prato *et al.* [40]. NCT00621140
1218.17	Linagliptin 5 mg Placebo;	523 177	Metformin	24	Taskinen *et al.* [41]. NCT00601250
1218.18	Linagliptin 5 mg Placebo;	792 263	Metformin + sulfonylurea	24	Owens *et al.* [42]. NCT00602472
1218.20	Linagliptin 5 mg Glimepiride 1–4 mg;	776 775	Metformin	104	NCT00622284
1218.23	Linagliptin 5 mg Linagliptin 10 mg Voglibose 0.6 mg Placebo;	159 160 162 80	None	26	NCT00654381
1218.35	Linagliptin 5 mg Placebo;	157 81	Sulfonylurea	18	NCT00819091
1218.36*	Linagliptin 5 mg Placebo;	628 627	Basal insulin	≥52	NCT00954447
1218.43	Linagliptin 5 mg Placebo;	67 63	None	52	NCT00800683
1218.46	Linagliptin 5 mg Placebo;	428 363	Metformin	24	NCT00798161
1218.50	Linagliptin 5 mg Placebo;	151 76	None	52	NCT00740051
1218.52^†^	Linagliptin 5 mg Placebo;	396 170	Metformin	54	NCT00915772
1218.61*	Linagliptin 5 mg Placebo;	183 89	Metformin + pioglitazone	24	NCT00996658
1218.62*	Linagliptin 5 mg Placebo;	447 44	Metformin	12	NCT01012037
1218.63*	Linagliptin 5 mg Placebo;	162 79	None	24	NCT01084005
1218.64*	Linagliptin 5 mg Placebo;	113 122	None	52	NCT01087502
1218.65*	Linagliptin 5 mg Placebo;	205 100	Metformin	24	NCT01215097
1218.66*	Linagliptin 5 mg Placebo;	200 99	None	24	NCT01214239
1218.75*	Linagliptin 5 mg Placebo;	101 115	None	24	NCT01194830

*Trials with prospective independent adjudication of hospitalization for CHF.

^†^Trial 1218.52 is an extension of study 1218.46 and was analyzed in conjunction with 1218.46, and therefore is not displayed as an individual study in other displays. CV, cardiovascular; CHF, congestive heart failure.

### Analysis population

Common inclusion criteria across the included trials were a diagnosis of T2DM, age ≥18 years, glycated hemoglobin (HbA1c) 7–10% entrance criterion in most studies, and body mass index (BMI) 20–45 kg/m^2^. In all studies, if deterioration in glycemic control occurred, rescue therapy could be initiated. In general, rescue therapy was initiated if glucose levels exceeded 240, 200, or 180 mg/dL (after an overnight fast) on 2 separate days during the first 12, 12–24, or >24 weeks, respectively. Data collected after initiation of rescue therapy were included in the analysis.

### Laboratory, BP, heart rate, and weight assessment

Changes from baseline in blood pressure (BP), heart rate, weight, and lipid parameters (including total cholesterol, low-density lipoprotein [LDL] cholesterol, high-density lipoprotein [HDL] cholesterol, and triglycerides) to the last available on-treatment measurement were assessed.

### CV event data collection and adjudication

Data on AEs were collected by the study investigators using electronic case report forms. During the pivotal phase 3 trials for linagliptin, a prospectively defined adjudication process was implemented to assess cardiac and neurological vascular events, including deaths, through 2 independent, blinded, external CECs, respectively, for cardiac events and neurological events. Adjudication for the event ‘hospitalization for CHF’ was introduced while the phase 3 program was in progress and, therefore, implemented for a limited number of studies (8 studies, involving 3314 patients [n = 2039 linagliptin and n = 1275 total comparators]; Table 2).

**Table 2:** **FDA custom MACE end point**

**The following MedDRA preferred terms (version 15.0) are included in the FDA custom MACE end point:**	
Myocardial infarction terms	Stroke terms
Myocardial Infarction terms	Basilar artery thrombosis
Acute myocardial infarction	Brain stem infarction
Coronary artery thrombosis	Brain stem stroke
Myocardial infarction	Brain stem thrombosis
Papillary muscle infarction	Carotid arterial embolus
Postprocedural myocardial infarction	Carotid artery thrombosis
Silent myocardial infarction	Cerebellar infarction
	Cerebral artery embolism
	Cerebral artery thrombosis
	Embolic stroke
	Haemorrhagic cerebral infarction
	Haemorrhagic stroke
	Haemorrhagic transformation stroke
	Ischaemic cerebral infarction
	Ischaemic stroke
	Lacunar infarction
	Lateral medullary syndrome
	Moyamoya disease
	Postprocedural stroke
	Stroke in evolution
	Thalamic infarction
	Thrombotic cerebral infarction
	Thrombotic stroke
	Wallenberg syndrome

FDA, Food and Drug Administration; MACE, major adverse cardiovascular event.

After identification of a trigger AE, a data package for the CEC was prepared. This data package contained patient profile information (based on the demographic and clinical safety data in the clinical trial database); all available electrocardiograms and reports on angiography scans, ultrasound, or duplex scans or echocardiography, as appropriate; all available laboratory data; and other relevant medical documents. Based on these data packages, either the cardiac or neurology CEC performed the adjudication of the trigger event, blinded to treatment allocation, and documented the adjudication result. Upon completion of a trial, these adjudicated events were collected and included in the clinical trial database.

In the additional analysis, occurrence of events suggestive of CHF (narrow MedDRA 15.1 SMQ cardiac failure) was assessed from the investigator-reported AEs. It should be noted that the number of events suggestive of CHF reported from the investigators does not take into account the adjudication status of these cases and is not limited to events leading to hospitalization.

### End points

The primary end point was time to the first occurrence of any components of the 4P-MACE composite. Secondary end points were composites of: (i) 3P-MACE; (ii) all adjudicated CV events, which included CV death, non-fatal MI, non-fatal stroke, unstable angina, stable angina, and transient ischemic attacks (TIAs); (iii) FDA-defined custom major adverse CV events (MACE), derived from 34 MedDRA preferred terms for stroke and MI (these terms are listed in Table 2). Tertiary end points were the individual adjudicated end points (as listed above) plus coronary revascularization procedures, hospitalization for CHF, stent thrombosis, and all-cause mortality.

### Statistical analysis

The analyses based on individual patient-level data in the treated set were defined as all patients who were randomized and received at least 1 dose of study medication. Changes in CV risk factors from baseline to last treatment were expressed descriptively. Overall incidences and incidence rates (per 1000 patient-years) were calculated for all end points in each of the pooled treatment groups within the treated set. The CV risk estimate after treatment with linagliptin in comparison with control treatment (and for 4P-MACE and CHF for placebo-controlled studies only) is expressed as HR for time to first event, using Cox proportional hazards model, with adjustments for treatment and study groups. Sensitivity analyses were performed to assess the impact of rescue medication on the analyses of the primary end point. Pre-specified subgroup analyses were performed to evaluate the incidence and incidence rates for the subgroup variables: age (≤50, 51 to <65, 65 to <75, and ≥75 years), gender, race, use of rescue therapy, occurrence of hypoglycemia, Framingham 10-year coronary heart disease (CHD) risk score (≤15% or >15%), renal function (estimated glomerular filtration rate [eGFR] according to Modification of Diet in Renal Disease [MDRD] formula), and microalbuminuria (albumin/creatinine ratio [ACR]).

The number of patients with symptoms suggestive of CHF, based on the MedDRA narrow SMQ Cardiac failure, was reported descriptively.

## Results

### Patient characteristics and drug exposure

Pooled data on adjudicated events were evaluated from 19 studies (17 placebo-controlled, 1 with placebo/active control, 1 with active control only), which included 9459 patients treated with at least 1 dose of study drug. In total, 5847 patients were treated with linagliptin; the majority of subjects received the 5-mg dose (n = 5687) (in study 1218.23, 160 patients received linagliptin 10 mg). There were 3612 subjects in the comparator group: 937 received active treatment (glimepiride 1–4 mg once daily [n = 775] or voglibose 0.2 mg 3 times daily [n = 162]) and 2675 received placebo. Depending on the design of the individual trial, some patients received their trial medication in addition to existing background glucose-lowering therapy. For the additional analysis of investigator-reported CHF, data from 24 placebo-controlled studies were evaluated (patients received linagliptin 5 mg or placebo as monotherapy, or in addition to background therapies). This evaluation included data from 8778 patients (5488 linagliptin- and 3290 placebo-treated patients). An overview of the included trials is provided in Tables 1 and 3.

**Table 3:** **Overview of additional clinical trials included in evaluation of congestive heart failure (CHF)**

**Study number**	**Treatment**	**Patients**	**Background**	**Follow-up (weeks)**	**Reference/NCT number**
1218.2	Linagliptin 1, 2.5, 5, 10 mg Placebo;	35 9	None	<2	Heise *et al.* [43]*.* NCT02183350
1218.3	Linagliptin, 2.5, 5, 10 mg Placebo;	61 16	None	4	Forst *et al.* [44]*.* NCT 02183415
1218.5	Linagliptin 0.5, 2.5, 5 mg Placebo;	170 67	None	30	Singh-Franco *et al.* [45]*.* NCT00328172
1218.6	Linagliptin 1, 5, 10 mg Placebo;	197 71	Metformin (most received ≥1500 mg/d)	12	Forst *et al.* [46]*.* NCT00309608
1218.37	Linagliptin 5 mg Placebo;	40 40	None	4	Rauch *et al.* [47]*.* NCT00716092
1264.3	Linagliptin 5 mg Pioglitazone 15, 30, 45 mg Linagliptin + pioglitazone;	105 284 274	None	Up to 54	NCT01183013

(Study 1218.20 in table was not included in the additional analysis, as this trial was not placebo-controlled).

The overall median drug exposure in the linagliptin group, for the main pooled analysis, was 175 days (range: 1–776 days). Corresponding exposure in the placebo group was 174 days (range: 1–707 days), in the active comparator group, 729 days (range: 3–804 days), and in the combined comparator group, 183 days (range: 1–804 days). The percentages of patients who received trial medication for at least 52 weeks were 31.6% for placebo, 31.0% for linagliptin, 69.7% for active control, and 42.3% for combined comparators. Cumulative exposure (patient-years) was 4133.7 for linagliptin and 3106.6 for total comparators.

Baseline characteristics were broadly similar across the treatment groups for the main pooled analysis (Table 4). More than half of the patients were white (58.2%, linagliptin group; 60.6%, total comparator group) and male (54.4% and 56.5%, respectively). The majority of patients were older than 50 years of age (78.0% and 78.4%, respectively). Mean (SD) BMI was 29.04 (5.19) mg/kg^2^ in the linagliptin group and 29.53 (5.19) mg/kg^2^ in the total comparator group. More than half of the cohort (56.8%) had a diagnosis of T2DM for >5 years, and the majority of patients (83.1%) had previously received at least 1 glucose-lowering drug. For the 19 trials used in the main analysis, the distribution of CV risk factors at baseline was generally similar between the treatment groups, with Framingham risk >15% recorded in 25% and 29% of patients in the linagliptin and total comparator groups, respectively. Corresponding frequencies of microalbuminuria (urinary albumin:creatinine ratio [UACR], >30 to ≤300 mg/g) at baseline were 24.3% and 22.7%, respectively.

**Table 4:** **Baseline demographics and clinical characteristics, including CV risk factors, of the main study cohort in the linagliptin safety analysis (of adjudicated events)**

	**Linagliptin (n = 5847)**	*Active comparators (n = 937)	**Placebo (n = 2675)**	**Total comparators (n = 3612)**
Gender, % of patients Male/female	54.4/45.6	62.5/37.5	54.3/45.7	56.5/43.5
Age, years	59 ± 11	60 ± 10	58 ± 11	59 ± 10
BMI, kg/m^2^	29.0 ± 5.2	29.5 ± 4.8	29.5 ± 5.3	29.5 ± 5.2
Race, % of patients				
White	58.2	70.3	57.2	60.6
Black	3.7	1.9	6.8	5.5
Asian	38.1	27.7	36.0	33.8
HbA1c, mmol/mol	65 ± 10	61 ± 9	67 ± 10	65 ± 10
HbA1c, %	8.1 ± 0.9	7.8 ± 0.9	8.3 ± 0.9	8.1 ± 0.9
FPG, mmol/L	9.2 ± 2.5	9.2 ± 2.2	9.3 ± 2.7	9.3 ± 2.6
Diabetes duration, % of patients				
≤1 year	13.4	8.4	14.5	13.0
1–5 years	31.7	38.8	27.3	30.3
>5 years	54.9	52.7	58.2	56.8
Previous oral glucose-lowering agents, % of patients				
None	16.4	9.6	19.4	16.8
1	43.2	64.1	39.0	45.5
2	39.3	26.1	40.0	36.0
≥3	1.1	0.1	2.1	1.6
Antidiabetic drugs at enrolment, % of patients				
Metformin only	31.8	60.1	20.7	31.0
Metformin + other antidiabetic agents	38.1	25.2	38.2	34.9
Sulfonylurea only	6.5	2.0	8.0	6.5
Sulfonylurea + other antidiabetic agents	0.7	1.0	0.6	0.7
Insulin only	3.8	0.0	9.3	6.9
Insulin + other antidiabetic agents	<0.1	0.0	0.0	0.0
CV risk factors, % of patients				
Metabolic syndrome^†^	46.5	67.7	46.6	52.1
Coronary artery disease	11.8	12.3	14.1	13.6
Cerebrovascular disease	3.6	4.2	4.9	4.7
Peripheral artery disease	3.1	3.3	4.2	4.0
Hypertension	63.9	73.0	65.7	67.6
Ex-/current smoker	23.0/14.4	29.7/15.8	22.4/13.4	24.3/14.0
Microalbuminuria, % (UACR >30 to ≤300 mg/g)	24.3	21.6	23.0	22.7
Renal function based on eGFR (MDRD formula),% of patients				
Normal (≥90)	43.5	43.3	41.1	41.7
Mildly impaired (60 to <90)	45.5	49.8	43.5	45.1
Moderately impaired (30 to <60)	8.9	6.8	10.8	9.7
Severely impaired (<30)	1.9	0.0	4.1	3.0
CV medication, % of patients				
Acetyl-salicylic acid	31.8	32.3	34.5	33.9
Antihypertensive	60.9	69.6	62.0	64.0
Lipid-lowering therapy	41.7	49.0	43.6	45.0
Any of the above	72.7	81.6	73.9	75.9
Framingham 10-year CV risk score				
Score, %	9.7 ± 8.2	11.7 ± 8.6	9.6 ± 8.4	10.2 ± 8.5
Score >15%, % of patients	24.5	38.1	25.8	29.0

*BMI*, body mass index; *CV*, cardiovascular; *eGFR*, estimated glomerular filtration rate; *FPG*, fasting plasma glucose; *HbA1c*, glycated hemoglobin; *MDRD*, Modification of Diet in Renal Disease; *UACR*, urinary albumin:creatinine ratio.

*Glimepiride (n = 775), voglibose (n = 162).

^†^International Diabetes Federation definition.

Values are mean ± SD, unless otherwise stated.

The baseline characteristics of the dataset used for the additional evaluation of CHF showed similar findings (Table 5).

**Table 5:** **Baseline demographics and clinical characteristics, including CV risk factors for the CHF analysis (of investigator-reported events)**

	**Linagliptin (n = 5488)**	**Placebo (n = 3290)**
Gender, % of patients Male/female	53.5/46.5	54.9/45.1
Age, years	58.2 ± 10.6	58.1 ± 10.6
BMI, kg/m^2^	29.3 ± 5.3	30.0 ± 5.4
Race, % of patients		
White	59.5	63.0
Black	4.3	7.4
Asian	36.2	29.6
HbA1c, mmol/mol	66.1 ± 9.8	66.1 ± 9.8
HbA1c, %	8.2 ± 0.9	8.2 ± 0.9
FPG, mg/dL	166.2 ± 45.7	168.2 ± 47.4
Diabetes duration, % of patients		
≤1 year	15.4	15.8
1–5 years	30.6	30.3
>5 years	53.9	53.7
Missing	0.1	0.2
Antidiabetes drugs at enrolment, % of patients		
None	21.7	25.6
1	38.9	39.1
2	38.1	33.5
≥3	1.2	1.7

*BMI*, body mass index; *CHF*, congestive heart failure; *CV*, cardiovascular; *FPG*, fasting plasma glucose; *HbA1c*, glycated hemoglobin; *MDRD*, Modification of Diet in Renal Disease.

Values are mean ± SD, unless otherwise stated.

Active treatment produced a greater reduction in HbA1c compared with placebo. For CV risk factors evaluated (total cholesterol, LDL cholesterol, HDL cholesterol, triglycerides, systolic and diastolic BP, heart rate, and body weight), there were no meaningful differences between linagliptin and placebo or total comparators (Table 6).

**Table 6:** **Change in CV risk factors from baseline to last treatment (treated set)**

**Mean (SEM)**	**Linagliptin (n = 5758)**	**Active comparators (n = 918)**	**Placebo (n = 2618)**	**Total comparators (n = 3536)**
Total cholesterol, mg/dL				
Baseline	187 (0.6)	186 (1.3)	187 (0.9)	187 (0.8)
Change from baseline	1 (0.5)	2 (1.1)	2 (0.7)	2 (0.6)
LDL, mg/dL				
Baseline	107 (0.5)	104 (1.1)	107 (0.7)	106 (0.6)
Change from baseline	1 (0.4)	4 (0.9)	1 (0.6)	1 (0.5)
HDL, mg/dL				
Baseline	47 (0.2)	48 (0.4)	47 (0.3)	47 (0.2)
Change from baseline	1 (0.1)	−1 (0.2)	1 (0.2)	1 (0.1)
Triglyceride, mg/dL				
Baseline	173 (1.8)	180 (4.1)	174 (2.9)	176 (2.4)
Change from baseline	−7 (1.5)	−8 (4.2)	−3 (2.6)	−5 (2.2)
HbA1c, %				
Baseline	8.1 (0.01)	7.8 (0.03)	8.3 (0.02)	8.1 (0.02)
Change from baseline	−0.7 (0.01)	−0.5 (0.03)	−0.3 (0.02)*	−0.3 (0.02)
Weight				
Baseline	79.5 (0.25)	83.8 (0.58)	81.1 (0.39)	81.9 (0.32)
Change from baseline	−0.1 (0.05)	1.3 (0.15)	0.1 (0.07)	0.4 (0.06)
Systolic BP (mmHg)				
Baseline	131 (0.2)	134 (0.5)	132 (0.3)	132 (0.3)
Change from baseline	−1 (0.2)	−1 (0.5)	−1 (0.3)	−1 (0.3)
Diastolic BP (mmHg)				
Baseline	78 (0.1)	80 (0.3)	78 (0.2)	79 (0.2)
Change from baseline	−1 (0.1)	−1 (0.3)	−1 (0.2)	−1 (0.2)
Heart rate, bpm				
Baseline	74 (0.1)	73 (0.4)	74 (0.2)	74 (0.2)
Change from baseline	1 (0.1)	−1 (0.3)	1 (0.3)	1 (0.2)

*BP*, blood pressure; *bpm*, beats per minute; *CV*, cardiovascular; *HbA1c*, glycated hemoglobin; *HDL*, high-density lipoprotein; *LDL*, low-density lipoprotein.

*Analysis includes data obtained after initiation of glycemic rescue.

### Adjudicated CV events

**Figure 1:**
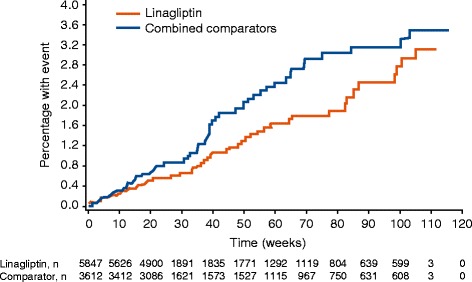
Time to first event (occurrence of any component of the 4P-MACE composite of CV death, MI, stroke, or UAP with hospitalization) for patients receiving linagliptin versus total comparators. *CV*, cardiovascular; *MI*, myocardial infarction; *UAP*, unstable angina pectoris.

**Figure 2:**
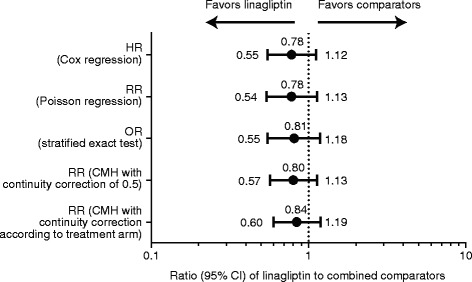
Risk estimates for primary composite CV end point with linagliptin versus total comparators based on various statistical models. *CI,* confidence interval; *CMH,* Cochran-Mantel-Haenszel; *CV,* cardiovascular; *HR,* hazard ratio; *OR,* odds ratio; *RR,* risk ratio.

Overall, 420 patients with AEs were identified from the pre-specified list of trigger events. A total of 60 (1.0%) primary components of 4P-MACE events were reported in the linagliptin group and 62 (1.7%) in the comparator group. The incidence rate of 4P-MACE was 13.4 events per 1000 patient-years for linagliptin-treated patients compared with 18.9 in the active comparator group (Table 7, Figure 1) with a Cox regression HR (Table 8) indicating no significant difference between the 2 treatment groups; HR, 0.78 (95% CI, 0.55–1.12) (Figure 2).

**Table 7:** **Incidence and incidence rates of primary, secondary, and tertiary end points**

	**Linagliptin (n = 5847)**		**Total comparators (n = 3612)**	
	**Incidence n (%)**	**Incidence rate (per 1000 years)**	**Incidence n (%)**	**Incidence rate (per 1000 years)**
Primary end points				
CV death, stroke, MI, or UAP with hospitalization	60 (1.0)	13.4	62 (1.7)	18.9
Secondary end points				
CV death, stroke, or MI	42 (0.7)	9.3	46 (1.3)	14.0
All major CV events	96 (1.6)	21.5	95 (2.6)	29.1
FDA-custom MACE	39 (0.7)	8.7	45 (1.3)	13.7
Tertiary end points				
CV death	11 (0.2)	2.4	8 (0.2)	2.4
Non-fatal MI	23 (0.4)	5.1	20 (0.6)	6.1
Non-fatal stroke	9 (0.2)	2.0	19 (0.5)	5.8
TIA	1 (0.02)	0.2	8 (0.2)	2.4
UAP with hospitalization	22 (0.4)	4.9	16 (0.4)	4.8
Hospitalization for CHF*	12 (0.6)	8.8	9 (0.7)	8.4
Total mortality	18 (0.3)	4.0	16 (0.4)	4.8

*CHF*, congestive heart failure; *CV*, cardiovascular; *FDA*, Food and Drug Administration; *MACE*, major adverse CV events; *MI*, myocardial infarction; *TIA*, transient ischemic attack; *UAP*, unstable angina pectoris.

*Includes data only from trials with prospective independent adjudication of hospitalization for CHF (n = 3314).

**Table 8:** **Risk for primary, secondary, and tertiary individual CV end points with linagliptin versus total comparators based on Cox proportional hazards model**

	**Cox HR (95% CI)**
Primary end point	
CV death, MI, stroke, or	0.78 (0.55–1.12)
UAP with hospitalization	
Secondary end points	
CV death, stroke, or MI	0.74 (0.49–1.13)
All major CV events	0.82 (0.61–1.09)
FDA-custom MACE	0.70 (0.45–1.08)
Tertiary end points	
CV death	1.04 (0.42–2.60)
Non-fatal MI	0.86 (0.47–1.56)
Non-fatal stroke	0.34 (0.15–0.75)
TIA	0.09 (0.01–0.75)
UAP with hospitalization	1.08 (0.56–2.06)
Hospitalization for CHF*	1.04 (0.43–2.47)
Total mortality	0.89 (0.45–1.75)

*CHF*, congestive heart failure; *CI*, confidence interval; *CV*, cardiovascular; *FDA*, Food and Drug Administration; *HR*, hazard ratio; *MACE*, major adverse CV events; *MI*, myocardial infarction; *TIA*, transient ischemic attack; *UAP*, unstable angina pectoris.

*Includes data only from 8 trials with prospective independent adjudication of hospitalization for CHF (n = 3314).

**Figure 3:**
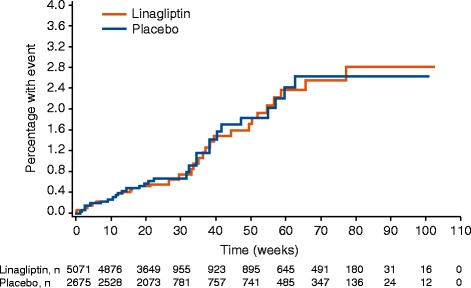
Time to first event (occurrence of any component of the 4P-MACE composite) for patients receiving linagliptin versus placebo.

In the placebo cohort of the overall group (ie, 18 of the 19 trials), 4P-MACE incidence rates (Figure 3) were 14.9 per 1000 patient-years for linagliptin (43 events) and 16.4 for total comparators (29 events), yielding an overall HR of 1.09 (95% CI, 0.68–1.75).

In the placebo cohort (n = 7746 patients) there was no signal for an increased risk of either all-cause or CV mortality with linagliptin therapy. All-cause mortality for linagliptin (2538 patient years exposure) versus placebo (1608 patient years exposure) was reported for 13 versus 11 patients, respectively; HR 0.81 (95% CI, 0.36–1.81). For CV mortality with linagliptin (2538 patient years exposure) versus placebo (1608 patient years exposure), 8 versus 6 deaths, respectively, were reported; HR 0.88 (0.30–2.55).

Subgroup analysis of the overall cohort showed that the risk estimate for the primary end point associated with linagliptin versus total comparators was not increased by the following factors: age, gender, race, use of rescue therapy, occurrence of hypoglycemia, Framingham 10-year CHD risk score (≤15% or >15%), renal function, microalbuminuria, or use of background medication (insulin and/or metformin) (Table 9).

**Table 9:** **Subgroup analyses of primary end point for linagliptin versus total comparators based on proportional Cox hazards model and CMH test**

	**Linagliptin,patients with events/total patients**	**Total comparators,patients with events/total patients**	**Cox HR (95% CI)**	**Incidence ratio (95% CI) CMH Test**
Age (years)				
≤50	5/1288	3/781	1.31 (0.31–5.58)	1.11 (0.47–2.63)
51 to <65	26/2817	26/1740	0.75 (0.43–1.30)	0.86 (0.52–1.41)
65 to 75	26/1418	29/902	0.78 (0.46–1.32)	0.84 (0.52–1.37)
≥75	3/324	4/189	0.63 (0.14–2.85)	0.88 (0.37–2.08)
Gender				
Male	42/3183	50/2039	0.69 (0.45–1.04)	0.73 (0.49–1.08)
Female	18/2664	12/1573	1.22 (0.58–2.55)	1.27 (0.68–2.36)
Race				
White	46/3405	49/2190	0.78 (0.52–1.17)	0.83 (0.56–1.23)
Black	4/215	1/200	3.92 (0.44–35.08)	1.50 (0.55–4.11)
Asian	10/2227	12/1222	0.59 (0.25–1.37)	0.76 (0.39–1.49)
Use of rescue medication				
No	43/5080	46/2824	0.70 (0.46–1.07)	0.75 (0.50–1.12)
Yes	17/767	16/788	1.10 (0.56–2.19)	1.11 (0.64–1.91)
Investigator-reported hypoglycemia				
No	48/5197	37/2918	0.86 (0.56–1.32)	0.97 (0.65–1.45)
Yes	12/650	25/694	0.79 (0.39–1.59)	0.78 (0.41–1.47)
Framingham 10-year CV risk score				
≤15%	20/3797	24/2438	0.65 (0.36–1.18)	0.76 (0.45–1.29)
>15%	36/1433	38/1046	0.85 (0.54–1.35)	0.91 (0.60–1.39)
Baseline microalbuminuria				
Normal (≤30 mg/g)	25/3610	25/2389	0.84 (0.48–1.47)	0.91 (0.56–1.50)
Elevated (>30 to ≤300 mg/g)	21/1248	21/771	0.75 (0.41–1.38)	0.83 (0.49–1.41)
High (>300 mg/g)	6/280	14/241	0.43 (0.16–1.11)	0.63 (0.31–1.26)

*CI*, confidence interval; *CV*, cardiovascular; *HR*, hazard ratio; *CMH test*, Cochran-Mantel-Haenszel test with treatment arm continuity correction.

**Figure 4:**
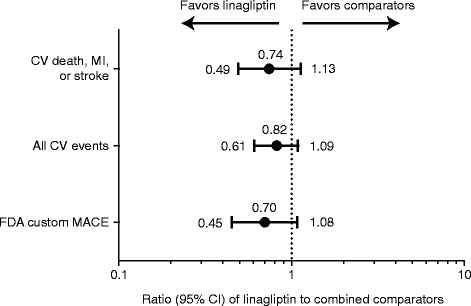
HR estimates for secondary composite CV end points with linagliptin versus total comparators based on Cox hazard model. *CI*, confidence interval; *CV*, cardiovascular; *FDA*, Food and Drug Administration; *HR*, hazard ratio; *MACE*, major adverse CV events; *MI*, myocardial infarction.

In line with the results for the primary end point, the incidence rates and the HRs for the secondary end points were similar for linagliptin and total comparators (Tables 7 and 8). The overall risk estimates were also similar for tertiary end points for linagliptin and total comparators (Table 7, Figure 4), with a significantly reduced HR for stroke and TIA favoring linagliptin compared with total comparators. For hospitalization for CHF, a small number of patients reported events (n = 21), and the overall risk estimate was similar for linagliptin (12 events; 2039 patients) and the total comparator group (9 events, 1275 patients), with a HR of 1.04 (95% CI, 0.43–2.47). This was also the case in a sensitivity analysis of the smaller treatment cohort, excluding data beyond 12 weeks from 1 trial (1218.64) in which 107 placebo-treated patients were switched to treatment with glimepiride. In this sensitivity analysis, the total number of adjudicated CHF events was 9 in the linagliptin group and 5 in the placebo group, yielding a non-significant HR of 1.29 (95% CI, 0.43–3.87).

In addition to the evaluation of hospitalization for heart failure in the 8 studies in which adjudication took place, further analysis of investigator-reported and non-adjudicated events from 24 placebo-controlled studies of linagliptin 5 mg versus placebo showed that events suggestive of heart failure were reported in 0.5% (n = 26) of linagliptin- and 0.2% (n = 8) of placebo-treated patients (Table 10). Among these events, those considered to be serious were reported in 0.3% (n = 16) of linagliptin- and 0.2% (n = 6) of placebo-treated patients.

**Table 10:** **Occurrence of adverse events suggestive of heart failure in 24 placebo-controlled trials of linagliptin**

	**Linagliptin**	**Placebo**
	**(n = 5488)**	**(n = 3290)**
Cardiac failure (narrow SMQ)*, n (%)	26 (0.5)	8 (0.2)
Acute pulmonary edema	1 (0.0)	1 (0.0)
Cardiac failure	6 (0.1)	2 (0.1)
Cardiac failure, acute	2 (0.0)	0 (0.0)
Cardiac failure, chronic	1 (0.0)	0 (0.0)
Cardiac failure, congestive	7 (0.1)	5 (0.2)
Cardiogenic shock	2 (0.0)	0 (0.0)
Cardiopulmonary failure	1 (0.0)	0 (0.0)
Left ventricular failure	6 (0.1)	0 (0.0)
Pulmonary edema	1 (0.0)	2 (0.1)
Right ventricular failure	1 (0.0)	0 (0.0)

*MedDRA*, Medical Dictionary for Regulatory Activities; *SMQ*, standardized MedDRA queries.

Data are based on MedDRA version 16.0.

*Heart failure data are a total of the narrow SMQ listed. Individual patients may have had >1 event.

## Discussion

This comprehensive pooled analysis evaluated patient-level data from 9459 subjects with T2DM who participated in 19 clinical trials. It represents a collective 4421.3 patient-years of exposure to linagliptin—approximately twice as many years of exposure as previous analyses. In line with the conclusion of the previous analysis, this new analysis also suggests that linagliptin does not increase CV risk or CV outcomes versus combined comparator therapies or versus placebo, in patients with T2DM [18]. Furthermore, the findings of this analysis also indicate that the CV risk profile was not influenced by a number of important factors associated with CV complications (age, gender, race, use of rescue medication, occurrence of hypoglycemia, Framingham CHD risk, renal disease, and microalbuminuria). The findings of the present analysis add to the existing evidence base for DPP-4 inhibitors, which shows the class to be generally well tolerated, but with less definitive evidence regarding CV safety [19].

To enable a relevant assessment of CV risk, studies evaluating the CV safety of glucose-lowering therapies should include patients at increased risk of CV events, such as those with relatively advanced disease, elderly patients, and those with some degree of renal impairment [11]. In the present analysis, baseline data on the Framingham risk status of participants indicated that about 30% of patients had a 10-year risk score of >15%, that approximately half of all patients had been diagnosed with T2DM for >5 years, and that around 13% of patients were older than 70 years of age. In addition, some degree of renal impairment (eGFR based on MDRD staging of <90 mL/min) was present in just over half of patients analyzed (56.3% in the linagliptin and 57.9% in total comparator groups, respectively), with nearly a quarter (24.3% and 22.7%, respectively) having microalbuminuria. Thus, a sizeable proportion of the analyzed population may be deemed to exhibit moderately increased CV risk. Indeed, the observed incidence of rates of 4P-MACE (13.4 events and 18.9 events per 1000 patient-years for linagliptin- and active comparator-treated groups, respectively) is slightly higher than would be predicted from the Framingham risk scores. This observation, combined with the large number of patients exposed to linagliptin and the higher number of CV events accrued compared with previous analyses, therefore, supports the validity of the findings.

As noted above, the incidence of CV events reported in this analysis, per 1000 patient-years of exposure, was 13.4 for linagliptin and 18.9 for total comparators. Other, similar, analyses of phase 3 studies have reported incidence rates for custom MACE ranging from 5.8 to 14.6 for sitagliptin, saxagliptin, or vildagliptin, and 9.0 to 14.1 for pooled comparators [20-22]. The most recent assessment of the CV safety of sitagliptin, based on pooled data from 25 double-blind studies, showed an incidence rate ratio, per 100 patient-years, of 0.83 (95% CI, 0.53–1.30) for the comparison of sitagliptin versus pooled comparators, and 1.01 (95% CI, 0.55–1.86) for sitagliptin versus placebo [23]. These findings are in line with the results of the present analysis. Although the previous analyses of the CV safety of DPP-4 inhibitors differ in their methods, the findings nonetheless support the hypothesis that, in general, DPP-4 inhibitor therapy is not associated with increased CV risk. This was also supported by the neutral outcomes on 3P-MACE of the prospective studies SAVOR-TIMI 53 and EXAMINE, conducted in patients with T2DM at high CV risk [12,13].

EXAMINE was designed to investigate the CV risk of alogliptin compared with placebo in patients with T2DM and recent acute coronary syndrome [13]. Similar rates of major CV AEs in addition to CV or all-cause mortality were reported for alogliptin- and placebo-treated patients. The SAVOR TIMI 53 trial evaluated the effects of saxagliptin on CV outcomes in patients with T2DM at high CV risk. No difference in the primary composite end point of CV death, MI, or ischemic stroke was found in saxagliptin- or placebo-treated patients [12]. It should be noted that both of these trials were relatively short in duration (median follow-up, respectively, 2.2 and 1.5 years) and included patients predominantly, or exclusively, with manifest CV complications, 2 important considerations when assessing the potential CV risk modulation of any compound [24,25].

The unexpected finding of an increased risk of CHF in these trials merits further careful evaluation [12,14,15]. A recent study of echocardiograms from 254 patients with T2DM and existing CHF (New York Heart Association [NYHA] class I to III), who received vildagliptin or placebo for 52 weeks, found that vildagliptin therapy did not change the cardiac ejection fraction (the primary end point of the study), and was not associated with worsening of CHF (confirmed by a blinded adjudication committee); worsening CHF occurred in 22 patients in the placebo group compared with 23 in the vildagliptin group [26]. However, patients taking vildagliptin, compared with those taking placebo, showed unexpected increases in left ventricular end-diastolic volume (*P* = .007), end-systolic volume (*P* = .06), and stroke volume (*P* = .002). The possible mechanisms underlying these observations are not fully understood. Two recent meta-analyses of available data from randomized clinical trials of DPP-4 inhibitors have indicated that these drugs could be associated with an increased risk of acute heart failure [27,28]. However, both meta-analyses included data from the SAVOR-TIMI 53 trial, the results of which strongly influence the overall findings. Furthermore, a new analysis of data from EXAMINE, presented at the Scientific Sessions of the American College of Cardiology in March 2014, indicated that alogliptin did not increase the risk of new-onset heart failure or the risk of readmission in patients with T2DM and a history of heart failure [29]. In the linagliptin clinical trial program, adjudication of hospitalization for CHF was implemented during phase 3b. Given the small number of reported cases of CHF (for the overall comparator analysis, n = 12 and n = 9; and for the comparison with placebo only, n = 9 and n = 5), the reported HR of 1.04 (95% CI, 0.43–2.47) in the overall comparator analysis, or 1.29 (95% CI, 0.43–3.87) in the placebo-only analysis must be interpreted with some caution. Similarly, in the additional analysis of investigator-reported AEs in 24 placebo-controlled studies, the occurrence of events suggestive of heart failure was low (0.5% [n = 26 of 5488] and 0.2% [n = 8 of 3290] for linagliptin- and placebo-treated patients, respectively), and was within the expected background incidence for this population. Therefore, this database is currently too small to allow firm conclusions to be made regarding the impact of linagliptin on the risk of heart failure. So far, there has been no explanation for the increase in risk of CHF observed in the trials mentioned above, and data are currently limited. However, it has been noted that trials with reported CV outcomes tend to include patients who are older, have a longer duration of T2DM, more CV risk factors, lower renal function, more comorbidities, and often treated with a greater number of antidiabetic drugs, including insulin, when compared with other studies of patients with T2DM [19]. These factors might be relevant in the identification of subpopulations who could be at increased risk of CHF or other CV outcomes with DPP-4 inhibitor therapy. Reassuringly, a review of safety data for DPP-4 inhibitors has demonstrated the safety and tolerability of these agents in fragile populations such as elderly patients and individuals with renal impairment [30]. Similarly, a recent pooled analysis of 6 clinical trials demonstrated the safety and tolerability of linagliptin in a vulnerable subpopulation of patients at high risk of renal or CV disease [31]. A recent systematic review and network meta-analysis of 10 clinical trials of DPP-4 inhibitors in patients requiring third-line therapy for T2DM showed no difference between these agents and placebo in the incidence of adverse events, including CVD [32]. This analysis thus provides further evidence of the safety and tolerability of DPP-4 inhibitors in patients with T2DM that is difficult to control.

The ongoing Trial Evaluating Cardiovascular Outcomes with Sitagliptin (TECOS), designed to test the hypothesis that sitagliptin added to usual diabetes care does not increase CV risk in patients with existing CVD (estimated mean trial duration, approximately 4 years) [33], will also provide an evaluation of DPP-4 inhibitor therapy over a longer period than SAVOR-TIMI 53 or EXAMINE, and should provide additional information on the CHF issue.

An interesting finding of the present pooled analysis is the significant reduction in cerebrovascular end points observed in linagliptin-treated patients, compared with the other groups, albeit limited by small number of observations. This finding would need to be further evaluated, in particular because similar results were not observed in the recent SAVOR-TIMI 53 and EXAMINE trials [12-14]. Whether linagliptin could offer benefits following stroke is another possible area for further research in light of preclinical findings that have demonstrated a glucose-independent neuroprotective effect of linagliptin in the diabetic mouse brain model, possibly as a result of neural stem cell proliferation [34]. Potential improvements in endothelial function with DPP-4 inhibition, as indicated in some studies, could have implications for cerebrovascular outcomes [35,36]. The impact of linagliptin on cerebrovascular events and post-stroke function is currently under investigation in 2 outcome trials, as discussed below.

As with all pooled analyses, the present analysis has several limitations; in particular, the relatively short and different durations of the included studies limit the extent of interpretations that can be made. Furthermore, despite a large cumulative patient exposure to linagliptin, individual patient exposure was for a maximum of 2.2 years, so the time available for the development of CV events, or modulation of CV risk, was limited. As might be expected, only a relatively small proportion of patients received triple therapy or insulin therapy at baseline, suggesting that a relatively limited number of patients in the study population had advanced T2DM. However, the robustness of the findings of the present study is supported by its pre-specified design, which incorporated independent prospective, blinded adjudication of CV events, and by the consistency of the results, both across the individual trials and across the different pools of results. However, none of the individual studies included in our analysis was powered or designed to assess CV risk or events.

Two ongoing CV outcome trials of linagliptin will provide a more definitive answer on the CV safety profile of linagliptin. One of these trials, the **CAR**diovascular **O**utcome Study of **LINA**gliptin versus Glimepiride in Patients with Type 2 Diabetes (CAROLINA^®^) [37,38] (NCT01243424) started in 2010 and has randomized 6041 patients with early T2DM and predominantly medium CV risk, to treatment with either linagliptin or glimepiride. CAROLINA^®^ is the first head-to-head CV outcome trial of a DPP-4 inhibitor versus active comparator that is sufficiently powered to demonstrate potential differences in CV events between treatment groups. CAROLINA^®^ will allow an assessment of the impact of long-term linagliptin therapy in a population at lower overall CV risk, with the possibility of demonstrating a CV benefit compared with the active comparator glimepiride.

The second CV outcome study, **CAR**diovascular Safety & Clinical outco**ME** with **LINA**gliptin (CARMELINA^®^), will compare the CV and renal safety of linagliptin versus placebo, when added to standard care in approximately 8300 patients with T2DM at high CV and renal compromise, and is the only large outcome study dedicated to the evaluation of tangible renal outcomes with a DPP-4 inhibitor in comparison with placebo. The CARMELINA^®^ trial was initiated in 2013 and results are expected between 2017 and 2018.

## Conclusion

This large patient-level pooled safety analysis of linagliptin supports previous findings that linagliptin is not associated with an increase in CV risk, compared with a pooled comparator group of placebo, glimepiride, or voglibose, in patients with T2DM, irrespective of background therapy. In this analysis, linagliptin has been shown to be effective in improving glycemic control in a broad range of patients with type 2 diabetes including elderly patients and those with renal impairment. The data presented here could help to reassure clinicians prescribing linagliptin to their patients that they do so without increasing CV risk. The ongoing CAROLINA^®^ and CARMELINA^®^ studies will provide definitive data on the CV safety of linagliptin.

## Abbreviations

ACR: Albumin/creatinine ratio
AE: Adverse event
BI: Boehringer Ingelheim
BMI: Body mass index
BP: Blood pressure
bpm: Beats per minute
CAROLINA: **CAR**diovascular Outcome Study of **LINA**gliptin versus Glimepiride in Patients with Type 2 Diabetes
CARMELINA: **CAR**diovascular Safety & Clinical outco**ME** with **LINA**gliptin
CEC: Clinical event committee
CHD: Coronary heart disease
CHF: Congestive heart failure
CI: Confidence interval
CMH: Cochran-Mantel-Haenszel
CV: Cardiovascular
CVD: Cardiovascular disease
DPP: Dipeptidyl peptidase
eGFR: Estimated glomerular filtration rate
FDA: US Food and Drug Administration
FPG: Fasting plasma glucose
HbA1c: Glycated hemoglobin
HDL: High-density lipoprotein
HR: Hazard ratio
LDL: Low-density lipoprotein
MACE: Major adverse CV event
MDRD: Modification of diet in renal disease
MedDRA: Medical dictionary for regulatory activities
MI: Myocardial infarction
NYHA: New York Heart Association
OR: Odds ratio
RR: Risk ratio
SAVOR TIMI 53: The Saxagliptin Assessment of Vascular Outcomes Recorded in Patients with Diabetes Mellitus – Thrombolysis in Myocardial Infarction 53 trial
SMQ: Standardized MedDRA queries
SU: Sulfonylurea
T2DM: Type 2 diabetes mellitus
TECOS: Trial evaluating cardiovascular outcomes with sitagliptin
TIA: Transient ischemic attack
UACR: Urinary albumin:creatinine ratio
UAP: Unstable angina pectoris
